# Depression among refugee youth in an outpatient healthcare center—prevalence and associated factors

**DOI:** 10.3389/fpsyt.2024.1367799

**Published:** 2024-04-19

**Authors:** Lea Schumacher, Jette Echterhoff, Areej Zindler, Dana Barthel

**Affiliations:** ^1^ Department of Medical Psychology, University Medical Center Hamburg-Eppendorf, Hamburg, Germany; ^2^ Outpatient Center GmbH, Refugee Outpatient Clinic, University Medical Center Hamburg-Eppendorf, Hamburg, Germany; ^3^ Department of Child and Adolescent Psychiatry, Psychotherapy, and Psychosomatics, University Medical Center Hamburg-Eppendorf, Hamburg, Germany

**Keywords:** refugee minors, associated factors, depression, PTSD, prevalence

## Abstract

**Background:**

Due to armed conflict and other crises, many children worldwide have to flee their home country and are, consequently, at a high risk for mental health problems.

**Objective:**

As the majority of previous research on refugee minors focused on post-traumatic stress disorder (PTSD), we aimed to assess the prevalence and risk factors for depression in a clinical sample of refugee youth.

**Methods:**

Data were collected during the standard diagnostic process in an outpatient refugee clinic in Germany. We assessed the prevalence of depression based on a diagnostic interview and investigated the association between age, gender, duration of flight, accompanying status, number of interpersonal traumatic experiences, residence status, and PTSD diagnosis with a depression diagnosis. More specifically, we conducted a Bayesian logistic regression with these associated factors as predictors and the presence of depression as the outcome. Additionally, we conducted a Bayesian network analysis including all these variables.

**Results:**

The majority of the 575 included refugee children were male (*n* = 423, 73.6%) and, on average, 15.1 years old (*SD* = 2.69). Nearly half of the children (*n* = 243, 42.3%) met the diagnostic criteria for depression, of which most also showed a comorbid PTSD diagnosis. We found strong evidence that age, gender, number of traumatic experiences, and a diagnosis of PTSD were related to depression. The network analysis indicated that only age, gender, and PTSD were directly associated to depression. Flight-related factors were only indirectly associated with depression due to their associations with number of traumatic experiences and PTSD diagnosis.

**Conclusion:**

The high prevalence of depression and its strong associations with PTSD suggest that refugee minors are likely to experience depressive symptoms which might develop from PTSD symptoms. This implies a need for monitoring depressive symptoms in refugee minors, especially when these have a PTSD diagnosis.

## Introduction

Exposure to war, armed conflicts, and persecution in many parts of the world have led to the flight of as many people as never before. The number of forcibly displaced people worldwide reached up to 103 million in mid-2022 (1). By the end of 2021, 36.5 million refugees, nearly half of the refugee population then, were children and adolescents under the age of 18 years (1). Refugee children and adolescents are likely to experience a variety of potentially traumatizing events in their home country, during the flight, and also in their host country (2, 3). These potentially traumatic experiences can lead to severe mental health problems (2). Besides post-traumatic stress disorder (PTSD), one of the most common mental disorders in refugee children and adolescents is depression. A recent systematic review and meta-analysis found an overall prevalence of 13.8% of depression in refugee minors (4). Another systematic review reported a prevalence rate of depression between 10.3% and 32.8% in refugee children, which is considerably larger than the prevalence rate of depression in the general population of children and adolescents (3). Refugee minors with mental health problems often met the criteria for more than one mental health disorder (5), but there is little research about the comorbidity of depression and PTSD in refugee children and adolescents. Nevertheless, Im and colleagues (6) found high rates of comorbidity among Somali refugees in Kenya, with estimates ranging from 21.6% to 28.8%. A recently conducted study examining trauma and levels of psychological distress among unaccompanied refugee minors found estimates ranging between 53.3% and 65.2% for a probable comorbidity of PTSD and depression diagnosis (7). In a small sample of Yazidi refugee minors, PTSD and depression were comorbid in 7.3% of the refugee children andadolescents (8). While several symptoms overlap between a PTSD and depression diagnosis, previous research indicated that both disorders constitute two distinct concepts (9–12). A high comorbidity for PTSD and depression remained even when disregarding overlapping symptoms, and the PTSD or depression symptoms were more strongly associated to symptoms included in the same diagnosis compared to symptoms included in the respective other diagnosis (11, 13). Still the comorbidity between both disorders might be due to relations between individual symptoms, i.e., the presence of specific PSTD symptoms might lead to the presence of depression symptoms or *vice versa* (13). Overlapping symptoms such as sleep problems as well as non-overlapping symptoms such as avoidance of thought seem to play a role for connecting depression and PTSD symptoms (13–15). Initial research indicated that PTSD might be a causal factor for developing subsequent depression; however, their relationship is likely to be complex and bi-directional (12, 16).

To better understand the mental health problems of refugee minors, factors influencing their mental health problems have been investigated. The association of gender with depression in refugee minors seems to be rather heterogeneous in previous literature (17, 18). Fazel and colleagues (18) reported in their review that approximately half of the studies found that girls had more depressive symptoms than boys, while other studies did not find such a gender-related difference. The systematic review of Daniel-Calveras and colleagues (17) also displayed such contradicting results. Furthermore, there seem to be an association between the age at migration and mental disorders (17). A higher age seems to be correlated with a higher burden of psychological problems (19) and with less changing symptoms of depression over time (20). Next to demographic variables, factors of the minors’ circumstances can also influence the likelihood of developing mental health problems. Most studies show that a larger number of traumatic experiences are related to more mental health problems (17). As a longer duration of flight might be associated with more traumatic events, flight duration might also impact mental health problems. However, so far, this was not explicitly studied. Furthermore, unaccompanied minors were more likely to experience higher levels of depression (21) and PTSD (22) compared to accompanied children and adolescents. Another factor possibly influencing the mental health of the refugee children and adolescents is the security of their residence status in the host country. In Germany, refugee minors receive a rather safe residence status (e.g., permission to stay in Germany for 3 years) if they obtain asylum or require refugee protection (23). In short, refugees receive asylum or refugee protection if they can prove that they are persecuted in their home country (24). Subsidiary protection for an initial period of 1 year is granted when the refugee can provide compelling reasons why they face serious harm from political or non-political actors in their home country (25). Current literature suggests that the residence status of refugee children and adolescents is associated with a PTSD diagnosis (26). A systematic review reported that refugee minors with only temporary residency had higher mental health problems compared to those with permanent residency (27). Strikingly, most previous evidence on risk factors in refugee minors relate to mental health problems in general or to PTSD, but hardly directly to depression.

Taken together, refugee minors show a higher prevalence of depression than minors in the general populations, and several factors seem to influence the likelihood of mental health problems in refugee minors. However, as pointed out by Kien and colleagues, the evidence for the prevalence of depression is limited for refugee minors due to risk of bias and inconsistency in previous studies (3). Furthermore, the majority of the current studies on risk factors for mental disorders in forcibly displaced children and adolescents refer primarily to PTSD as an outcome and depression more incidentally (17). Thus, no clear picture on the prevalence and risk factors for depression in refugee minors has emerged yet. One issue that further hampers the understanding of risk factors for depression is that the effect of each individual factor has been investigated in isolation to other factors. This ignores that risk factors also influence each other and together impact the development of depression—for example, unaccompanied children seem to experience significantly more traumatic events than accompanied refugee minors (17) and a longer flight duration is also probably related to a higher number of traumatic events. In this, the interplay between risk factors can be seen as a complex system of interacting variables, and it needs to be investigated as such.

Therefore, the aims of this cross-sectional study were threefold: (1) reporting the prevalence of depression and its comorbidity with PTSD in young forcibly displaced patients of an outpatient healthcare center for refugees in Hamburg, Germany, (2) investigating a variety of associated factors as predictors for a diagnosis of depression in these refugee minors, and (3) exploring the associations among all assessed factors and depression as a complex interacting system with a network analysis.

## Materials and methods

### Participants and procedure

Data were collected between October 2016 and December 2022 during the standard diagnostic process of the outpatient healthcare center for refugee children of the University Medical Center Hamburg-Eppendorf. This outpatient center provides psychiatric, psychotherapeutic, and psychosocial care for refugee children and adolescents that seek treatment for psychological problems. All children and adolescents that approached the outpatient clinic participated in a standardized diagnostic process. Minors that showed excessive psychological distress, current psychotic symptoms, or signs of intoxication entered a stabilization phase before they continued with the diagnostic process. The diagnostic procedure included the assessment of sociodemographic variables (e.g., gender, age, and residence status) and screening questionnaires for PTSD symptoms (CRIES-8 (28, 29)) and depression symptoms (PROMIS (30)). Additionally, modules of the structured clinical diagnostic interview MINI-KID (31) were implemented to diagnose depression and PTSD according to the ICD-10. Finally, the refugee minors were asked how often they experienced different potentially traumatic events. All assessments were administered in an interview with a psychologist. The assessments were conducted with the help of a language mediator, in the native language of the patient or in German. Children and adolescents were only included if their parents or, when unaccompanied, their legal guardians provided informed consent and ethical approval was received from the Chamber of Psychotherapists in Hamburg (05/2017-PTK-HH).

### Measures

All screening and diagnostic instruments used in the diagnostic process were reported elsewhere (32). Only instruments used in the current analysis are described in detail in the following. Relevant measures for the present study were diagnosis of depression and PTSD and the following related factors: gender, age, accompanying status during flight, duration of flight, residence status, and number of interpersonal traumatic events.

#### Diagnosis of depression and PTSD

Two modules of the of the German version of the “Mini-International Neuropsychiatric Interview for Children and Adolescents” (MINI-KID) were used to assess a depression and PTSD diagnosis. The MINI-KID is a structured diagnostic interview assessing psychological disorders in children and adolescents according to the DSM-IV and ICD-10. It showed very good reliability and validity as well as very good to excellent test–retest and interrater reliability (33).

The MINI-KID module A was used for the assessment of depression diagnosis (31). It assesses the presence and severity of a major depressive episode with 11 items. The three main criteria of depression according to ICD-10 were measured: depressed mood, loss of interest/joy, and loss of energy. In addition, other common symptoms, e.g., difficulty in concentration, sleeping problems, and change in appetite, were assessed. In the present study, two further items were added to allow the diagnosis of a major depressive episode with psychotic symptoms (F32.3) and to differentiate a depressive episode from grief and from depressive symptoms caused by drugs. For the current analysis, the depression variable was dichotomized in 0 = no depressive episode or mild depressive episode (F32.0) and 1 = moderate depressive episode (F32.1) or major depressive episode without psychotic symptoms (F32.2).

PTSD diagnosis was assessed with the MINI-KID module K. The items of module K assessed feelings related to a stressful life event in the month before the interview using dichotomous response categories (yes/no) per item. Experiencing and re-experiencing a traumatic event was assessed with single items, while avoidance was assessed with seven items and hyperarousal with five items. A PTSD diagnosis was present if the items on experiencing and re-experiencing a traumatic event were answered with “yes” in combination with at least two symptoms of avoidance and one symptom of hyperarousal.

#### Associated factors for a diagnosis of depression

The following sociodemographic variables were assessed by self-report: age, gender (0 = male, 1 = female), number of interpersonal traumatic experiences, accompanying status during flight (0 = accompanied, 1 = unaccompanied), flight duration (in days), and residence status (0 =secure, 1 = insecure). The number of traumatic experiences was measured by a study-specific questionnaire that contains items derived and modified from other questionnaires (32). The instrument assessed the frequency of the occurrence of 12 different specific interpersonal traumatic experiences with “never”, “once”, “several times”, and “not clear”, and the life phase in which the traumatic event occurred (home country, flight, and host country/Germany). These traumatic experiences included, for example, exposure to war, neglect, separation from family/close friends, and different forms of abuse. To calculate a sum score for the interpersonal traumatic events, answers were coded with the following scores: “never” = 0, “once” = 1, and “several times” = 2. Accompanying status during flight was defined as the presence of a parent or a legal guardian during the flight. The residence status was dichotomized in 0 = secure (residence permit) and 1 = insecure (temporary resident permit, exceptional leave to remain, and church asylum were not regarded as a secured residence status). It is worth noting that we did not consider home country as an associated factor since it seems a rather indirect approximation for describing the previous experiences of the children. Especially when considering the long time frame of this study (2016–2022), the home country of the refugee minor might have had a different impact depending on the situation in the respective country at a given time point.

### Statistical analysis

Missing data of the previously described risk factors and diagnosis of depression were imputed before the analysis with the EM algorithm. The prevalence of depression diagnosis and its comorbidity with PTSD, a Bayesian logistic regression, and a Bayesian cross-sectional network analysis were calculated. A sensitivity analysis with complete cases was also computed. The data was analyzed with R version 4.42 using the packages brms (34) and BDgraph (35).

#### Prevalence of depression and comorbidity with PTSD

The prevalence of depression diagnosis and its 95% confidence interval were calculated. The 95% confidence interval was calculated with SE= √(*p* (1 - *p*)/*n*). Additionally, the comorbidity with a PTSD diagnosis was examined.

#### Bayesian logistic regression

A Bayesian logistic regression was calculated to test the association between several associated factors with a depression diagnosis. The factors age, gender, duration of flight, number of traumatic experiences, accompanying status, residence status, and diagnosis of PTSD were included in the model as predictors and diagnosis of depression as outcome. We choose weakly informative priors for all parameters using a normal distribution with a mean of 0 and a standard deviation of 2, which grant more weight to small values. The regression was conducted with the R package brms. The model was estimated with Markov chain Monte Carlo sampling with 10 chains, each with 5,000 iterations. For each predictor, we calculated a Bayes factor (BF_01_) that indicates how much evidence the data provides that the predictor is larger than zero.

#### Bayesian network analysis

Network analysis is a methodological approach that aims to investigate the interaction between variables. Networks display the conditional dependencies between all included variables, i.e., how variables are associated while taking all other variables into account. Bayes network analysis uses Bayesian inference to estimate the associations among variables, given prior information and the observed data. This method allows to quantify the certainty of each estimated parameter. A Bayesian mixed graphical model was estimated with the R package BDgraph (35). Age, duration of flight, and number of traumatic experiences entered the network analysis as continuous variables, while gender, accompanying status (flight), residence status, PTSD diagnosis, and depression diagnosis were added as categorical variables. A noninformative prior on the network structure was determined, i.e., a prior edge inclusion probability of 0.5 was specified. For the prior of the precision estimate, a G-Wishart distribution with three degrees of freedom was defined. Edges, i.e., associations between individual variables, with a posterior inclusion probability of at least 50%, were displayed in the network models. Edge weights show how strongly two variables were connected. For each estimated edge weight, a Bayes factor (BF) was calculated, which suggests how probable the respective connection is present or absent in the network. A BF_10_ of one indicates equal evidence for edge inclusion and exclusion (36). In the current study, we considered a BF higher than 10 as strong evidence for the presence of an edge/association and a BF between 3 and 10 as weak evidence for this edge/association.

## Results

### Sample description

Sample characteristics of the *N* = 575 recruited participants are displayed in **Table 1**. Data on all associated factors was available for 318 refugee minors. Missing data for each analyzed variable ranged from one missing data point for gender to 178 missing data points for duration of flight, with a median of 69 missing data points for each variable. The majority of the sample was male (*n* = 423, 73.6%), and the participants were, on average, 15.1 years old (*SD* = 2.69, range = 7–20 years). As displayed in **Table 1**, most refugee minors came from Afghanistan (*n* = 223, 38.8%), were unaccompanied during flight (*n* = 306, 53.2%) and in Germany (*n* = 286, 49.7%), and had an uncertain residence status (*n* = 328, 57.0%). Nearly one quarter of the sample were currently living in a supervised youth living (*n* = 131, 22.8%). The participants had a mean number of 13.6 of traumatic experiences ranging from 0 to 38 experiences, and the mean duration of the flight was 349.5 days (range = 0–3,285 days). About half met the criteria for a PTSD diagnosis (*n* = 301, 52.35%).

**Table 1:** Sociodemographic characteristics of the sample (*N* = 575).

	n	%
Gender		
Male	423	73.57
Female	151	26.26
NA	1	0.17
Home country		
Afghanistan	223	38.78
Syria	111	19.30
Iran	39	6.78
Eritrea	32	5.57
Somalia	26	4.52
Guinea	25	4.35
Egyptian	9	1.57
Other	106	18.43
NA	4	0.70
Accompanying status (flight)		
Accompanied	256	44.52
Unaccompanied	306	53.22
NA	13	2.26
Residence status		
Secure	215	37.39
Insecure	328	57.04
NA	32	5.57
Housing condition (Germany)		
Supervised youth living (full-time)	131	22.78
Housing shelter	103	17.91
Supervised youth living (part-time)	95	16.52
With parents	93	16.71
Own apartment	50	8.70
Primary care facility	31	5.39
Other	55	9.57
NA	17	2.96

N = 575. The participants were, on average, 15.09 years old (SD = 2.69, range = 7–20 years).

#### Prevalence of depression and comorbidity with PTSD

Nearly half of the refugee minors met the criteria of a depression diagnosis (*n* = 243, 42.26%). More specifically, 2% (CI: 1%–3%) met the criteria for a mild depressive episode, 11% (CI: 8%–13%) for a moderate depressive episode, and 31% (CI: 28%–35%) for a severe depressive episode. **Table 2** shows the comorbidity between a diagnosis of moderate to severe depression and a PTSD diagnosis. Most participants had no depression and no PTSD diagnosis or a comorbid PTSD and depression diagnosis (about one-third each). Only a few refugee minors (*n* = 49, 8.5%) had a depression diagnosis and no comorbid PTSD diagnosis.

**Table 2:** Comorbidity depression diagnosis with PTSD diagnosis.

		No/mild depression	Moderate/severe depression	Sum
		*N* (%)	*N* (%)	*N* (%)
No PTSD	*N* (%)	195 (33.91)	49 (8.52)	244 (42.43)
PTSD	*N* (%)	137 (23.83)	194 (33.74)	331 (57.57)
Sum	*N* (%)	332 (57.74)	243 (42.26)	575 (100)

Diagnosis criteria of the ICD-10 were applied.

PTSD, post-traumatic stress disorder.

### Bayesian logistic regression

The results of the Bayesian logistic regression are displayed in **Table 3**. The multicollinearity between predictors was checked with bivariate correlations and the variance inflation factor (VIF), indicating a maximal correlation between predictors of 0.54 and a maximum VIF of 1.62. Significant predictors for a depression diagnosis were age, gender, number of traumatic experiences, and a diagnosis of PTSD, i.e., these predictors had 95% probability of influencing the likelihood of a depression diagnosis. More specifically, the regression analysis showed that older participants had a 1.23 higher chance to suffer from a depression than younger participants (OR = 1.23; 95% CI = 1.11–1.36, BF_01_ = Inf). Furthermore, female refugee minors were twice as likely to receive a depression diagnosis than male refugee minors (OR = 1.91; 95% CI = 1.23–3.00, BF_01 _= 605.06). The number of traumatic events increased the likelihood of a depression diagnosis with 1.04 (OR = 1.04; 95% CI = 1.01–1.07, BF_01 _= 201.02). A PTSD diagnosis had the largest impact on the probability of a depression diagnosis, as patients with a PTSD diagnosis were nearly four times more likely to receive a depression diagnosis than patients without a PTSD diagnosis (OR = 3.85, 95% CI = 2.45–6.07, BF _01_= Inf). The rather high Bayes factor for each of these predictors indicated that we found strong evidence that these predictors are larger than zero. We found somewhat strong evidence that duration of flight and accompanying status during the flight were not related to the likelihood of a depression diagnosis (BF_01_ of 0.06 and 0.13, respectively). The included predictors explained 21.14% of the variance in depression diagnosis (*R* ^2^ Bayes).

**Table 3:** Results of the logistic regression analysis.

Predictors	OR	95% CI	BF_01_
Age	1.23	1.11–1.36	Inf
Gender (reference = male)	1.91	1.23–3.00	605.06
Duration of flight (in days)	1	1.00–1.00	0.06
N traumatic experiences	1.04	1.01–1.07	201.02
Accompanying status (reference = accompanied)	0.75	0.47–1.20	0.13
Residence status (reference = secure)	1.07	0.69–1.65	1.62
PTSD	3.85	2.45–6.07	Inf

OR, odds ratio; CI, credibility interval; BF, Bayes factor; PTSD, post-traumatic stress disorder.

### Bayes network analysis

**Figure 1:**
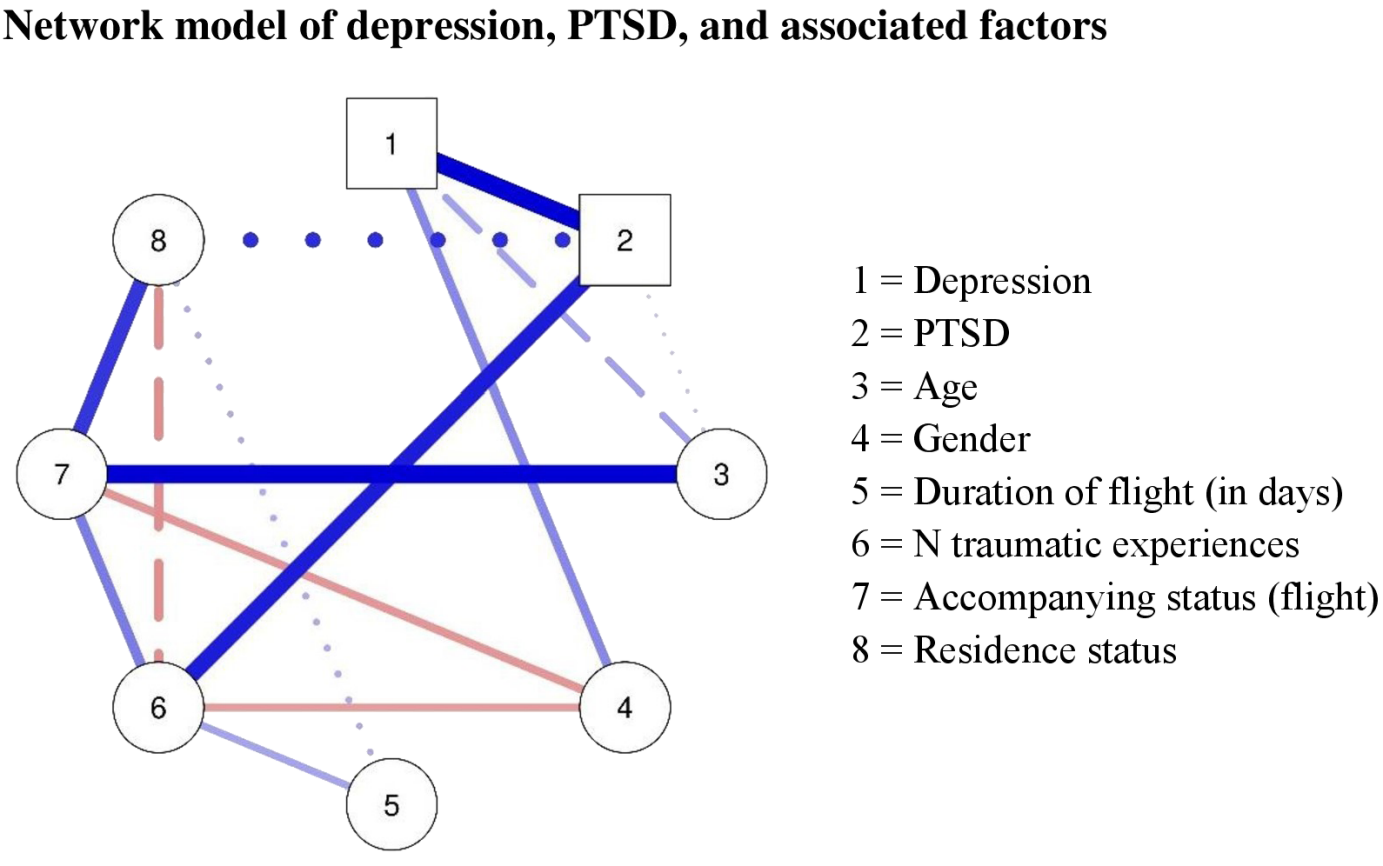
Network model of depression, PTSD, and associated factors. Red connections represent negative associations, while blue associations represent positive associations; thickness indicates the strength of the association. A strong evidence for the presence of an association is displayed with a solid line (BF < 10), a weak evidence for an association with a dashed line (BF > 3 < 10), and insufficient evidence (BF < 3) with dotted lines. PTSD, post-traumatic stress disorder; BF, Bayes factor.

**Figure 1** shows the cross-sectional network including PTSD diagnosis, depression diagnosis, and all associated factors. Depression had a positive connection with age with weak evidence to be present and a positive association with gender with strong evidence to be present, showing again that older age and female gender were associated with a higher likelihood of depression. The two diagnosis variables depression and PTSD were strongly positively associated, and the association showed strong evidence to be present. Furthermore, PTSD was positively linked to the number of traumatic experiences, and the connection had strong evidence of presence. The other factors were also highly associated between each other. The number of traumatic experiences was positively associated with duration of flight and with accompanying status, meaning that minors with a longer flight duration and unaccompanied minors had experienced more traumatic events. Additionally, being unaccompanied was related with older age and insecure residence status. Furthermore, the network showed negative associations between gender and accompanying status and between gender and number of traumatic experiences, which indicate that male individuals were more likely to be unaccompanied and had less traumatic experiences than females. Evidence plots of the network analysis can be found in **Supplementary Figure S1**.

### Sensitivity analysis with complete cases

As a sensitivity analysis, the prevalence of the depression diagnosis and its comorbidity with a PTSD diagnosis, the Bayesian logistic regression, and the Bayesian network analysis were calculated with complete cases only. The results of the sensitivity analysis are displayed in the **supplementary material** (**Supplementary Tables S1**-**S3**, **Supplementary Figure S2**) and showed no contradiction to the results of the main analysis.

## Discussion

As refugee minors are likely to experience a variety of mental health problems but most knowledge on mental health in refugee minors relate to PTSD, we aimed to assess the prevalence and associated factors for depression in refugee children and adolescents. Furthermore, we assessed how individual factors interact with each other to gain insight into how these factors might directly or indirectly lead to depression. We found that nearly half of this clinical sample met the diagnostic criteria for depression and about half met the criteria for a PTSD diagnosis. Furthermore, about one-third of the young patients experienced comorbid depression and PTSD. Our analysis found strong evidence that older age, female gender, a higher number of traumatic experiences, and a PTSD diagnosis increased the likelihood of meeting the criteria for a moderate or severe depressive episode. With the help of network analysis, we could show that depression was only directly related to age, gender, and PTSD and that flight-related factors were mainly associated to each other and with a PTSD diagnosis.

The observed prevalence of depression is substantially higher than in previous investigations (3, 4), which is not surprising given the clinical nature of this sample (our analyses included children and adolescents that approached the outpatient clinic because of psychological distress). Still, it emphasizes that refugee minors are at a high risk of developing depression and that there is a considerable need for appropriate treatment. The current study also strikingly showed that depression and PTSD are highly comorbid in refugee youth, as the majority of the sample either had both a depression and PTSD diagnosis or no depression and no PTSD diagnosis. This finding aligns with previous research indicating that depression and anxiety disorders are highly comorbid (37). The finding that older age and female gender are related to depression diagnosis aligns well with research on non-refugee children (38). Similarly, our study could confirm previous literature on the importance of the number of traumatic experiences for experiencing mental health problems (17). With more interpersonal traumatic events experienced, the refugee children were more likely to experience a depressive episode. The strongest predictor of a depression diagnosis was a PTSD diagnosis. This indicates that the activation of PTSD symptoms might have also led to the activation of depression symptoms. Previous studies already showed that depression and PTSD symptoms were strongly linked (14, 15). These findings suggest that PTSD symptoms might lead to the activation of depressive symptoms. Therefore, it might be advisable to also monitor depression symptoms in refugee youth that experience PTSD. However, the pathways between PTSD and depression are likely to be complex and may vary between patient groups (11, 37). Thus, more research on the relation between depression and PTSD is needed.

The network analysis could shed some light on how the different associated factors interacted with each other and directly or indirectly impacted the likelihood of experiencing depression. Interestingly, all environmental factors that related to the fact that children and adolescents fled their home country were only indirectly related to depression. While duration of flight, number of traumatic experiences, accompanying status during flight and residence status were related to each other, we found only evidence for the association between number of traumatic experiences and PTSD, which, in turn, was highly related to depression—that is all environmental risk factors were only connected with depression *via* the number of traumatic events and PTSD. This indicates that in refugee youth flight-related factors seem to influence the likelihood of a PTSD diagnosis which then was associated to depression. Some risk factors might act indirectly through other risk factors on the likelihood of developing depression—for example, female gender, being unaccompanied, and a longer duration of flight were associated with a higher number of traumatic events. This emphasized that risk factors form a complex system of interacting variables, and to promote our understanding of these, we need to investigate their associations simultaneously.

The current study is limited by the fact that we could only investigate cross-sectional associations; therefore, the results can only offer a hypothesis on causal relations between the investigated variables. Furthermore, as all included children and adolescents were recruited at one outpatient center in Germany, future research needs to investigate if such results generalize to other refugee minor samples. Additionally, this study assessed a clinical sample of refugee minors that sought treatment in an outpatient clinic. Thus, the current sample is not representative of the general population of refugee minors in Germany. Finally, the logistic regression indicated that only 21% of the variance in depression were explained by the included variables. Thus, other variables might also play an important role for depression in refugee youth—for example, daily stressors such as worries about family members back in their home countries, perceived discrimination, economic concerns, and others. Still by investigating a large sample and using robust Bayesian analyses, we could gain insights into a highly relevant topic: the mental health of refugee children and adolescents. If future studies replicate our findings, this will indicate that refugee minors with a PTSD diagnosis should be also monitored for depressive symptoms. The next step would be to provide a suitable treatment in order to stop the PTSD symptoms from expanding to depressive symptoms. As our results showed that certain associated factors seem to influence the mental health of refugee minors indirectly, various risk factors and their associations should be considered, as these also might have indirect associations with mental health problems. Most importantly, it seems that a considerable number of refugee minors suffer from depression, and this should not be forgotten when researching and treating the mental health of refugee minors.

## Data availability statement

The raw data supporting the conclusions of this article will be made available by the authors, without undue reservation.

## Ethics statement

The studies involving humans were approved by Psychotherapeutenkammer Hamburg. The studies were conducted in accordance with the local legislation and institutional requirements. Written informed consent for participation in this study was provided by the participants’ legal guardians/next of kin.

## Author contributions

LS: Conceptualization, Formal analysis, Methodology, Project administration, Writing – original draft, Writing – review & editing. JE: Data curation, Formal analysis, Methodology, Writing – original draft, Writing – review & editing. AZ: Conceptualization, Funding acquisition, Resources, Writing – review & editing. DB: Conceptualization, Data curation, Funding acquisition, Methodology, Project administration, Supervision, Writing – review & editing.
