# Supplementary material for: Depression among refugee youth in an outpatient healthcare center—prevalence and associated factors
Source: Front Psychiatry. 2024 Apr 19;15:1367799. doi: 10.3389/fpsyt.2024.1367799 (PMC11067052; doi:10.3389/fpsyt.2024.1367799)
Supplement: Supplementary file 1 [file DataSheet_1.pdf]

Supplemental Material

This document provides the supplemental material for the manuscript ´ Depression among refugee youth in an outpatient health care center – prevalence and associated factors´.

Figure S1

Evidence plots main analysis

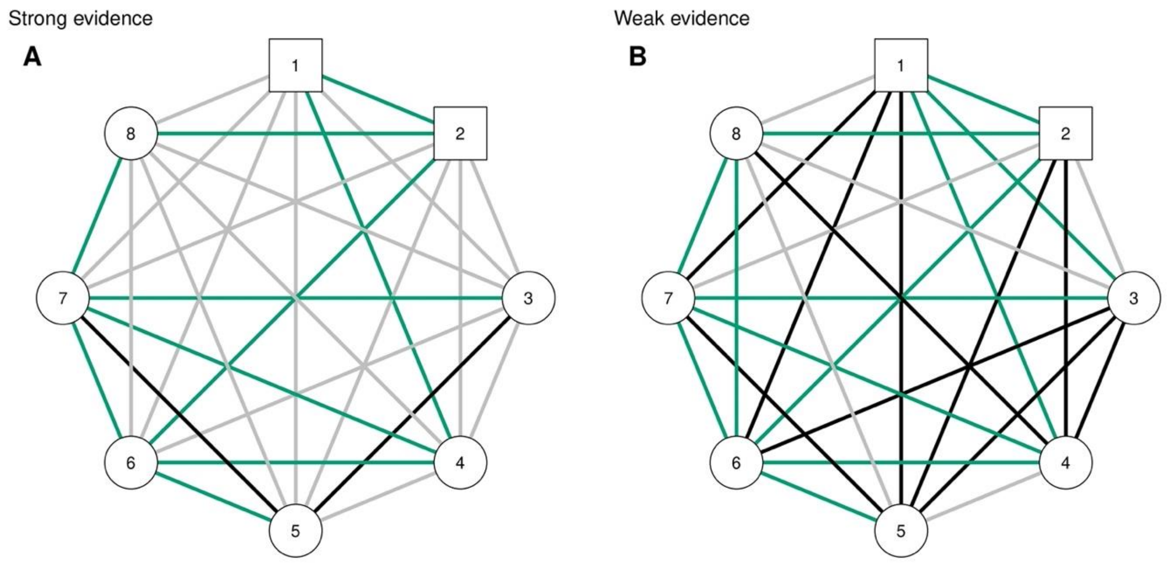

Note. 1 = Depression; 2 = PTSD; 3 = Age; 4 = Gender; 5 = Duration of flight (in days); 6 = N traumatic experiences; 7 = Accompanying status (flight); 8 = Residence status

Figure S1 shows evidence plots from the main analysis. Circles represent the risk factors and the squares the PTSD and depression diagnosis. Green relations represent edges with evidence for presence, whereas black connections represent edges with evidence of absence.

Table S1

Prevalence of depression sensitivity analysis

Depressive episode	Prevalence	95 % CI
Mild depressive episode	0.02	0.01 - 0.04
Moderate depressive episode	0.13	0.09 - 0.16
Severe depressive episode	0.33	0.28 - 0.38

Note. CI = Credibility interval, LL = Lower level, UL = Upper level

Table S2

Comorbidity depression diagnosis with PTSD diagnosis

		No/mild depression	Moderate/severe depression	Sum
		N (%)	*N (%)*	*N (%)*
No PTSD	*N (%)*	101 (31.0)	27 (8.3)	128 (39.2)
PTSD	*N (%)*	76 (23.3)	122 (37.4)	198 (60.7)
Sum	*N (%)*	177 (54.3)	149 (45.7)	326 (100)

Note. PTSD = Post-traumatic stress disorder. Diagnosis criteria of the ICD-10 were applied.

Table S3

Results of the logistic regression analysis

Predictors	OR	95 % CI	BF_{01}
Intercept	0.00	0.00 - 0.12	
Age	1.15	1.02 - 1.31	88.49
Gender	1.82	1.01 - 3.28	44.2
Duration of flight (in days)	1.00	1.00 - 1.00	0.88
N traumatic experiences	1.04	1.00 - 1.07	41.38
Accompanying status	0.95	0.50 - 1.79	0.79
Residence status	0.95	0.54 - 1.64	0.73
PTSD	4.35	2.43 – 7.89	Inf

Note. OR = Odds ratio, CI = Credibility interval, BF = Bayes factor, PTSD = Post-traumatic stress disorder

Figure S2

Network model of risk factors, depression, and PTSD sensitivity analysis

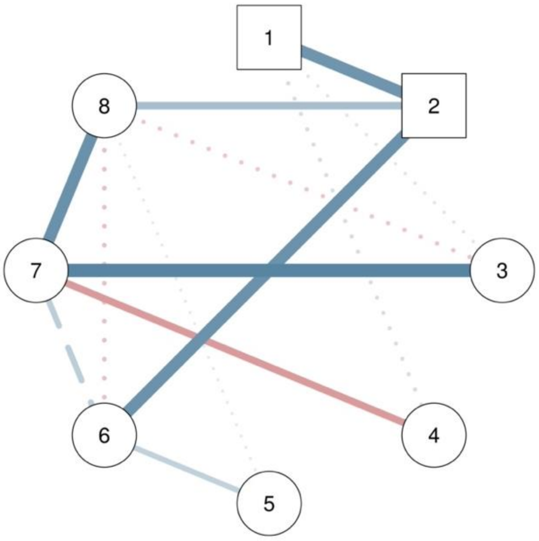

1 = Depression
2 = PTSD
3 = Age
4 = Gender
5 = Duration of flight (in days)
6 = N traumatic experiences
7 = Accompanying status (flight)
8 = Residence status

Note. Red connections represent negative edges, while blue associations represent positive edges, thickness indicates the edge weight, e.g., how strongly two nodes are connected. Strong evidence for the presence of an edge is displayed with a solid line (BF < 10), weak evidence for edges with a dashed line (BF > 3 < 10), and insufficient evidence (BF < 3) with dotted edges. BF = Bayes factor.

Figure S3

Evidence plots sensitivity analysis

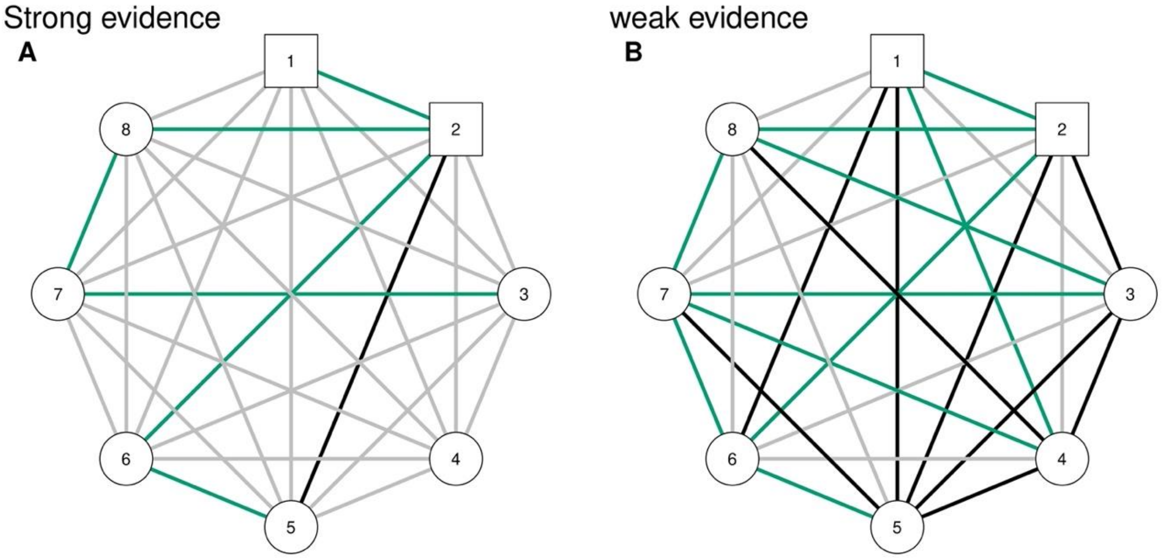

Note. 1 = Depression; 2 = PTSD; 3 = Age; 4 = Gender; 5 = Duration of flight (in days); 6 = N traumatic experiences; 7 = Accompanying status (flight); 8 = Residence status

Figure S3 shows evidence plots from the sensitivity analysis. Circles represent the risk factors and the squares the PTSD and depression diagnosis. Green relations represent edges with evidence for presence, whereas black connections represent edges with evidence of absence.
